# Development of Resveratrol-Loaded Herbal Extract-Based Nanocomposites and Their Application to the Therapy of Ovarian Cancer

**DOI:** 10.3390/nano8060384

**Published:** 2018-05-31

**Authors:** Suyeong Nam, Song Yi Lee, Wie-Soo Kang, Hyun-Jong Cho

**Affiliations:** 1 College of Pharmacy, Kangwon National University, Chuncheon, Gangwon 24341, Korea; swim@kangwon.ac.kr (S.N.); heymush@kangwon.ac.kr (S.Y.L.); 2 Department of Bio-Health Technology, College of Biomedical Science, Kangwon National University, Chuncheon, Gangwon 24341, Korea; kangwiso@kangwon.ac.kr

**Keywords:** *Angelica gigas* Nakai, anticancer activities, nanocomposite, resveratrol, ovarian cancer

## Abstract

Resveratrol (RSV) and the ethanol extract of *Angelica gigas* Nakai (AGN Ex)-based nanoparticles (NPs) were prepared using the nanocrystal concept. AGN/RSV NPs with 224 nm hydrodynamic size, unimodal size distribution, and negative zeta potential values were developed with the emulsification and solvent evaporation techniques. The crystalline properties of AGN Ex and RSV were transformed during the emulsification and solvent evaporation processes, thus, AGN NPs and AGN/RSV NPs exhibited amorphous states. AGN/RSV NPs held up their initial hydrodynamic size after 24 h of incubation in serum-included media. Sustained release profiles (for 5 days) of decursin (D) and decursinol angelate (DA) (the representative markers of AGN Ex) and RSV were observed at normal physiological pH (pH 7.4). In ovarian cancer (SKOV-3) cells, although AGN/RSV NPs showed a lower cellular entry rate rather than AGN NPs, the cellular accumulated amount of AGN/RSV NPs was similar with that of AGN NPs after 4 h of incubation. The antiproliferation efficiency of AGN/RSV NPs group was significantly higher than the AGN Ex, AGN NPs, and AGN NPs + RSV groups in SKOV-3 cells. AGN/RSV NPs can be one of the promising candidates for therapeutic nanoplatforms against ovarian cancers.

## 1. Introduction

Recently, many delivery approaches of drug cargos have been tried for cancer therapy [1,2,3,4,5]. The main purpose of those strategies is to elevate therapeutic efficacies and minimize toxicities by selective tumor targeting. In particular, nanocarriers have been attracted for the precise delivery of drug cargos to the malignant tumor region via intravenous administration [6,7]. Due to the specialized anatomical structures of tumor tissue (for example, leaky vasculature and immature lymphatic system), the distribution of nanocarriers with certain properties (for example, particle size and surface charge) in tumor tissue may be improved via an enhanced permeability and retention (EPR) effect [8,9]. However, the EPR effect-related passive tumor targeting strategy has intrinsic limitations. Therefore, active tumor targeting strategies (that is, the attachment of targeting ligands onto nanocarriers) have been developed to improve tumor targeting efficiency [10,11,12].

Together with small synthetic chemicals and biologics, single ingredients or whole extracts from the natural products have been used for the therapy of cancers [13,14,15]. Delivery of those natural products-derived active components can show equivalent therapeutic efficacies and reduce side effects, compared to small synthetic chemicals and biologics. Particularly, phytomedicines can be complementary and alternative therapeutic approaches to the typical anticancer agents. Among diverse phytomedicines for cancer prevention and therapy [16,17,18], the pharmacological effects of single components and extracts of *Angelica gigas* Nakai (AGN) have been widely investigated [19]. The main components of AGN, such as decursin (D) and decursinol angelate (DA), and its extracts have anticancer potentials in several types of cancers, such as breast, blood, cervical, colon, lung, oral, ovarian, prostate, and skin cancers [20,21,22,23,24,25,26]. In our previous studies [22,24,26], AGN extract and AGN particles have shown anticancer activities in breast, buccal, and cervical cancers.

The multifarious physicochemical properties of all ingredients included in the ethanol extract of AGN (AGN Ex) may hamper their delivery to tumor tissue. For the simultaneous delivery of diverse components of AGN Ex, single nanocarriers considering their different physicochemical characteristics should be designed. In our previous reports [24,26,27,28,29], several pharmaceutical technologies (that is, hot-melt extrusion, microemulsion, electrospraying/electrospinning, and emulsification-solvent evaporation) have been introduced to prepare the formulations of AGN powder or AGN Ex. Of note, polymer matrix-free AGN nanocomposites (NCs) were fabricated by introducing the nanocrystal preparation protocol [24]. The diverse ingredients of AGN Ex may act as matrices for the preparation of nanoparticles, thus, other pharmaceutical additives were not included except for the small amount of stabilizer. The minimal use of pharmaceutical excipients may avoid their toxicities after intravenous administration, thus, the feasibility for the clinical application of developed AGN NCs will be increased. In this study, resveratrol (RSV) was incorporated into AGN nanoparticles (NPs) to raise the anticancer activities against ovarian cancers. RSV can be found in the skin of blueberries, grapes, and raspberries. It has been used as one of the dietary supplements and it has shown a preventing effect against cancers, heart diseases, and stroke [30,31]. Due to its poor water-solubility, numerous pharmaceutical formulations have been developed for its therapeutic applications [32,33,34]. It is expected that RSV-loaded AGN NPs (AGN/RSV NPs) may exert anticancer activities of each component (that is, AGN Ex and RSV) thus, AGN/RSV NPs may provide elevated therapeutic potentials rather than AGN NPs. Herein, the particle characteristics, drug release patterns, cellular uptake efficiency, and antiproliferation efficacies of AGN/RSV NPs were investigated.

## 2. Materials and Methods

### 2.1. Materials

AGN was acquired from the market located in Pyeongchang (Korea). Poly(vinyl alcohol) (PVA, molecular weight: 30–70 kDa) and 1,1′-dioctadecyl-3,3,3′,3′-tetramethylindocarbocyanine perchlorate (DiI) were purchased from Sigma-Aldrich Co. (St. Louis, MO, USA). RSV and Tween 80 were obtained from Tokyo Chemical Industry Co. Ltd. (Tokyo, Japan). Standard samples of D (purity: ≥95%) and DA (purity: ≥75%) were purchased from the Korea Promotion Institute for Traditional Medicine Industry (Gyeongsan, Korea). Phosphate buffered saline (PBS), RPMI 1640, fetal bovine serum (FBS), and penicillin-streptomycin were obtained from Gibco Life Technologies, Inc. (Carlsbad, CA, USA). SKOV-3 cells were acquired from the Korean Cell Line Bank (Seoul, Korea). The other chemicals were analytical grade and used without further purification.

### 2.2. Preparation and Characterization of NPs

AGN Ex was obtained from AGN by the reported methods [24,26,27,28,29]. In short, AGN was dried at 55 °C for 1 day. Dried AGN was immersed in ethanol (EtOH) and they were incubated for 2 h at 80 °C. EtOH was then eliminated by the drying process and AGN Ex was obtained.

AGN NPs and AGN/RSV NPs were prepared by emulsification and solvent evaporation process [24,35,36]. For fabricating AGN NPs, AGN Ex (30 mg) was solubilized in dimethyl sulfoxide (DMSO, 0.5 mL) and dichloromethane (2.5 mL) was added to that solution. In case of AGN/RSV NPs, AGN Ex (30 mg) in DMSO (0.5 mL) and RSV (10 mg) in dichloromethane (2.5 mL) were mixed. Each organic phase was then dripped to PVA solution (0.5%, *w*/*v*; 30 mL). That emulsion was homogenized with an ultrasonic processor (VC-750; Sonics & Materials, Inc., Newtown, CT, USA) and the organic solvents were removed by stirring for 1 h. Hardened NCs were collected after centrifugation at 16,100× *g* for 30 min. The pellet of NPs was dispersed in distilled water (DW) and 3% (*w*/*v*) sucrose, as a cryoprotectant, was dissolved in that dispersion prior to lyophilization.

The encapsulation efficiency values of D/DA (in AGN NPs) and D/DA/RSV (in AGN/RSV NPs) were quantitatively determined by the high-performance liquid chromatography (HPLC) system as reported [24,26,27,32]. AGN Ex was dissolved in methanol (MeOH) at 1 mg/mL and standard samples of AGN Ex were prepared by serial dilution with MeOH. The sum of the D and DA contents, by comparing with those in AGN Ex, were used for calculating their encapsulation efficiency values in NPs. Freeze-dried NPs were dispersed in DW at 5 mg/mL and it was further diluted with MeOH for determining encapsulation efficiency values of AGN NPs and AGN/RSV NPs. D and DA contents were analyzed by the HPLC system (Jasco, Tokyo, Japan) consisted of an automatic injector (AS-2050 Plus), a pump (PU-2089 Plus), and a UV/Vis detector (UV-1575). The mobile phase was composed of DW including 0.2% formic acid (A) and acetonitrile containing 0.2% formic acid (B). Its gradient program was established as follows: (1) A:B = 70:30 (*v*/*v*) at 0 min; (2) A:B = 60:40 at 15 min; (3) A:B = 50:50 at 30 min; (4) A:B = 25:75 at 40 min; (5) A:B = 70:30 at 45 min; (6) A:B = 70:30 at 50 min; and (7) stop at 55 min. A reverse phase C18 column (250 mm × 4.6 mm, 5 μm particle size; Kinetex, Phenomenex, Torrance, CA, USA) was connected to the HPLC system for the quantitative determination of D and DA in AGN. The absorbance values were read at 329 nm. The flow rate was fixed at 1 mL/min and 10 μL of sample was injected into the HPLC system. Linearity was presented in a 1–500 μg/mL concentration range of AGN Ex. According to the reported method [32], the encapsulation efficiency of RSV in AGN/RSV NPs was also measured by HPLC system (Jasco, Tokyo, Japan) composed of an automatic injector (AS-2050 Plus), a pump (PU-2089 Plus), and an UV/Vis detector (UV-1575). For the preparation of the mobile phase, 10 mM potassium phosphate buffer (pH 6.8) and MeOH (50:50, *v*/*v*) were mixed. A reverse phase C18 column (250 mm × 4.6 mm, 5 μm particle size; Gemini, Phenomenex, Torrance, CA, USA) was used for the analysis of RSV. Freeze-dried AGN/RSV NPs were dispersed in DW at 5 mg/mL and were further diluted with MeOH prior to injection (at 20 μL injection volume) into the HPLC system. The flow rate was maintained at 1 mL/min during the HPLC analysis. The absorbance of RSV was monitored at 303 nm.

The hydrodynamic size and polydispersity index were measured by dynamic light scattering (DLS) method and zeta potential value was detected by the laser Doppler method at 5 mg/mL concentration in DW according to the instruction of manufacturer (ELS-Z1000; Otsuka Electronics, Tokyo, Japan).

The shape of AGN/RSV NPs dispersion was observed by an energy-filtering transmission electron microscope (EF-TEM). The AGN/RSV NPs dispersed in DW was loaded onto the copper grids with films and the droplet was stained with phosphotungstic acid (2%, *w*/*v*). Samples were washed with DW and the water content was removed under the air stream. The morphology of NPs was observed using an EF-TEM (LEO 912AB OMEGA; Carl Zeiss, Oberkochen, Germany).

The XRD data of AGN NPs and AGN/RSV NPs were obtained using the D8 ADVANCE with the DAVINCI model (Bruker AXS GmbH, Karlsruhe, Germany). CuKα-radiation (1.5418 Å) was used in the 5–45° 2θ range at 40 mA (current) and 40 kV (voltage). The step size was set at 0.02° and the scan speed was fixed at 0.5 sec/step.

### 2.3. In Vitro Stability Test of NPs

In vitro particle stability of AGN/RSV NPs was assessed after incubating in DW, 50% (*v*/*v*) FBS, and PBS (pH 7.4). The aliquot of dispersion of AGN/RSV NPs (10 mg/mL) in DW was mixed with the equivalent volume of each medium, such as DW, FBS, and PBS (pH 7.4). After incubating for 1, 2, 4, and 24 h at 37 °C, the hydrodynamic size of AGN/RSV NPs in each medium was monitored by the described DLS method.

### 2.4. Drug Release Test

The release behaviors of D, DA, and RSV from NPs were tested in the aqueous buffer (PBS, pH 7.4). For the maintenance of sink condition, Tween 80 (0.3%, *w*/*v*) was dissolved in the release medium (PBS, pH 7.4). AGN NPs or AGN/RSV NPs (6 mg) were dispersed in DW (0.15 mL) and they were loaded into the dialysis tube with 14 kDa molecular weight cut-off (mini GeBA-flex dialysis tube, Gene Bio-Application Ltd., Kfar Hanagide, Israel). That tube was then immersed in the release medium (30 mL) and it was incubated at 37 °C with 50 rpm speed. The release medium (0.2 mL) was collected and the fresh medium (0.2 mL) was supplemented at 4, 24, 48, 72, 96, and 120 h. The released amounts of D, DA, and RSV from NPs were calculated by the aforementioned HPLC methods.

### 2.5. Cellular Uptake Studies

The cellular accumulation pattern and intracellular location of developed NPs were evaluated in SKOV-3 cells. For the detection of fluorescence signals in cells, DiI (as a fluorescence dye) was incorporated into the AGN NPs and AGN/RSV NPs. For preparing DiI-loaded NPs, DiI (1 mg) was dissolved in dichloromethane with or without RSV. Other steps for the preparation of NPs were same with the described method in Section 2.2.

RPMI 1640 containing 10% (*v*/*v*) FBS and 1% (*v*/*v*) penicillin (100 U/mL) and streptomycin (0.1 mg/mL) was used for the culture of SKOV-3 cells at 37 °C in a humidified 5% CO_2_ atmosphere. After accomplishing 70–80% confluency, SKOV-3 cells were seeded in the 6-well plates at 6.0 × 10^5^ cells per well density and they were incubated for 1 day at 37 °C. Cells were treated with the dispersion of NPs (at 1 µg/mL DiI concentration) and they were incubated for 1 h and 4 h. SKOV-3 cells were washed with PBS (pH 7.4) and they were detached from the cell culture plate. Then, the cell pellets were resuspended in PBS including 2% (*v*/*v*) FBS. The fluorescence intensity-cell count profiles were acquired with a FACSCalibur fluorescence-activated cell sorter (FACS^TM^) installed with CellQuest (Becton Dickinson Biosciences, San Jose, CA, USA).

Cellular distribution of DiI-loaded NPs was observed through confocal laser scanning microscopy (CLSM) imaging. SKOV-3 cells (at 1.0 × 10^5^ cells per well (1.7 cm^2^ surface area per well) density) were seeded in the culture slide (BD Falcon, Bedford, MA, USA) and they were incubated for 1 day at 37 °C. DiI-loaded NPs (at 2 μg/mL DiI concentration) were applied to the cells and they were incubated for 1 h and 4 h at 37 °C. Those cells were washed with PBS (pH 7.4) at least thrice and formaldehyde solution (4%, *v*/*v*) was used for the fixation of cells. 4′,6-Diamidino-2-phenylindole (DAPI) included VECTASHIELD mounting medium (H-1200; Vector Laboratories, Inc., Burlingame, CA, USA) was applied to cells for staining the nuclei of cells and preventing fluorescence quenching. Fluorescence signals in cells were monitored by CLSM (LSM 880; Carl-Zeiss, Thornwood, NY, USA).

### 2.6. Antiproliferation Assay

The antiproliferation activities of RSV (RSV solution), AGN Ex (AGN Ex solution), AGN NPs, AGN NPs + RSV, and AGN/RSV NPs were evaluated by the colorimetric assay. SKOV-3 cells (at 2.0 × 10^3^ cells per well density) were seeded in the 96-well plate and incubated for 1 day. AGN Ex and RSV groups were prepared as a solution type. In case of AGN NPs, AGN NPs + RSV, and AGN/RSV NPs, each sample was dispersed or suspended in the cell culture media. AGN Ex, AGN NPs, AGN NPs + RSV, and AGN/RSV NPs (1–100 µg/mL concentration of AGN Ex) and RSV (1–100 µg/mL) were treated to cells and they were incubated for 72 h at 37 °C. Then, each sample was removed and CellTiter 96 Aqueous One Solution Cell Proliferation Assay Reagent (Promega Corp., Madison, WI, USA) was applied to the cells. The absorbance of each sample was detected at 490 nm by using a microplate reader (SpectraMax i3, Molecular Devices, Sunnyvale, CA, USA). Cell viability was calculated by comparison with the values of the control (no treatment) group.

### 2.7. Statistical Analysis

All experiments were repeated at least three times and the experimental data are shown as the mean ± standard deviation (SD). Two-tailed *t*-test and analysis of variance (ANOVA) were used for the statistical analysis of experimental data.

## 3. Results and Discussion

### 3.1. Preparation and Physicochemical Characteristics of NCs

**Figure 1:**
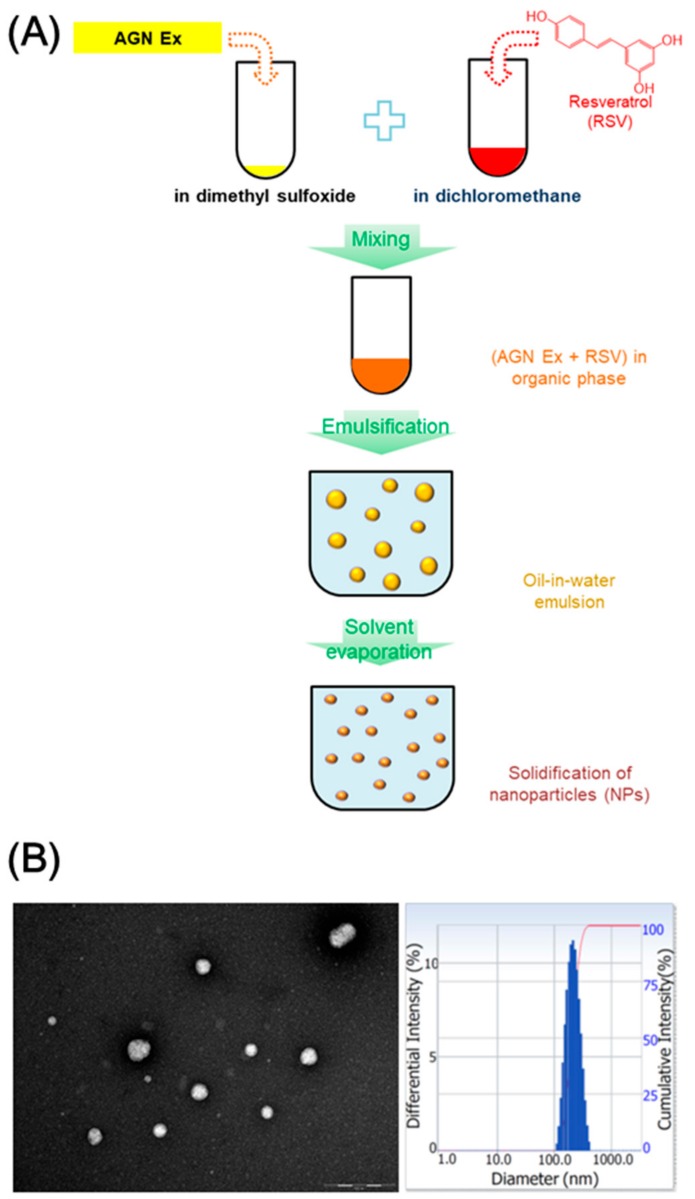
The fabrication and particle characterization of ethanol extract of *Angelica gigas* Nakai and resveratrol (AGN/RSV) nanoparticles (NPs). (**A**) The preparation method of AGN/RSV NPs; (**B**) The image of TEM (scale bar: 500 nm) and particle size distribution histogram of AGN/RSV NPs.

RSV-loaded AGN NPs were made by means of the modified emulsification-solvent evaporation method [24]. In our previous report [24], AGN nanoformulation was prepared without the use of polymer matrices by adopting the nanocrystal preparation protocol. AGN Ex and stabilizer (that is, PVA) were used for the development of AGN nanoformulation and PVA was almost removed during the preparation process. In this investigation, RSV was incorporated to AGN NPs to enhance anticancer activities in ovarian cancer cells. During the preparation process of AGN/RSV NPs, AGN Ex and RSV were dissolved in DMSO and dichloromethane, respectively, considering their solubility property (Figure 1A). The organic phase including AGN Ex and RSV was then added to the aqueous phase (that is, PVA solution). The oil-in-water emulsion was then stirred to eliminate the organic solvents and hardened nano-sized particles were obtained. AGN Ex and RSV can be contained in the single nanocarriers (AGN/RSV NPs in this study) and they could deliver those ingredients simultaneously to the cancer cells.

As shown in Table 1, the AGN NPs group exhibited a 221 nm hydrodynamic size, 0.16 polydispersity index, and −18.8 mV zeta potential values. During the fabrication process of AGN nanoformulation, 2% PVA solution was used as an aqueous phase in our previous study [24]. In this investigation, 0.5% PVA solution was used and it did not significantly alter the hydrodynamic size, size distribution, and surface charge of NPs. In addition, after RSV loading into the AGN NPs, they show comparable hydrodynamic size (224 nm mean diameter), particle size distribution (0.18 polydispersity index), and surface charge (−16.5 mV zeta potential) values, compared with AGN NPs. The observed hydrodynamic size seems to be appropriate for the EPR effect-related passive tumor targeting [8,9,37]. The narrow size distribution of developed AGN/RSV NPs may indicate the absence of micron-size aggregates when they are dispersed in the aqueous environment (Figure 1B). The round shape of AGN/RSV NPs, as well as their narrow size distribution, was also observed in TEM image (Figure 1B). The incorporation of RSV in AGN NPs did not significantly alter the surface charge of NPs. RSV was successfully incorporated into AGN NPs with similar encapsulation efficiency values of D and DA. Considering the observed particle properties of AGN/RSV NPs, they might be suitable for efficient tumor-selective delivery after intravenous administration [37].

**Figure 2:**
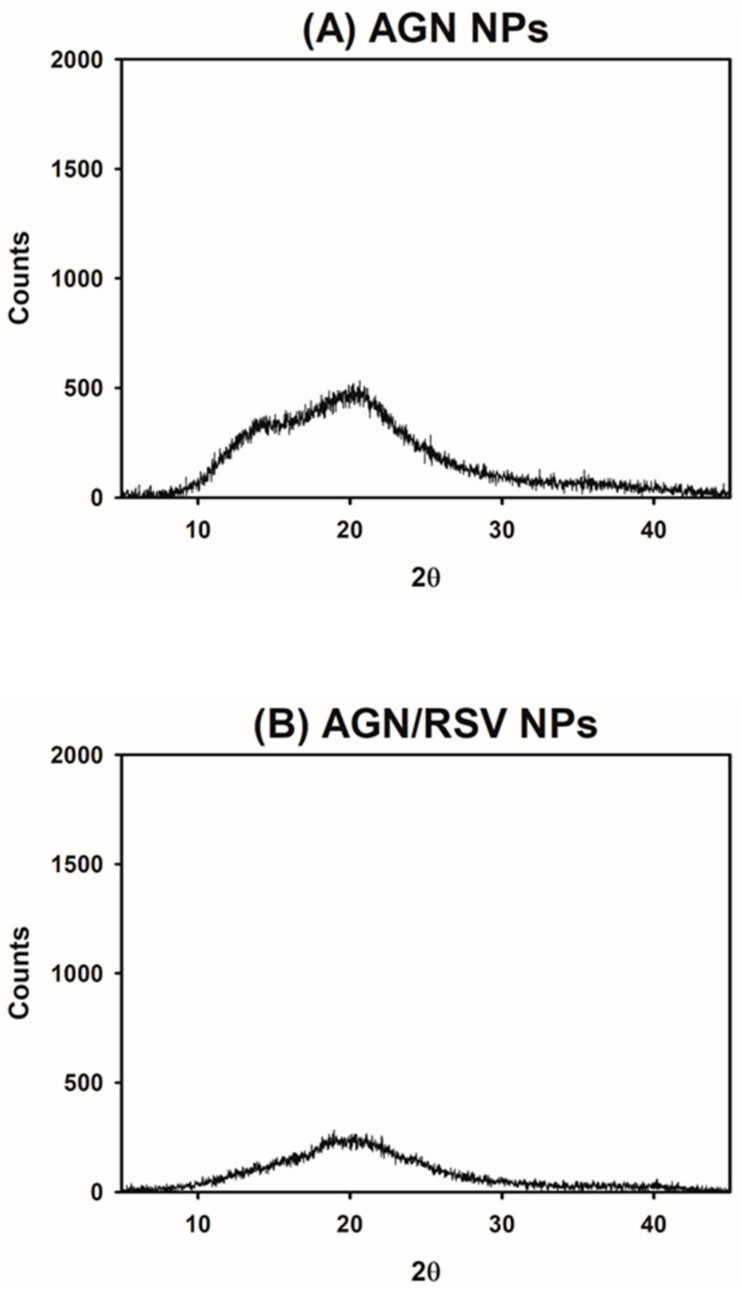
The XRD analysis data of NPs. Counts values of (**A**) AGN NPs and (**B**) AGN/RSV NPs, according to 2θ, are presented.

The solid states of developed AGN-based NPs were investigated by XRD test (Figure 2). The crystalline state of AGN Ex was already demonstrated in the previous study [27]. In contrast, sharp peaks are rarely shown in the profile of AGN NPs group, indicating their amorphous state (Figure 2A). The crystalline to amorphous transition of AGN components seemed to occur during the emulsification and solvent evaporation processes. The crystalline property of RSV was already revealed in our previous report [32]. However, it was altered to the amorphous state after its loading into the AGN NPs, as shown in the profile of AGN/RSV NPs (Figure 2B). As presented in the result of AGN Ex, the fabrication process might influence its amorphization. The amorphization of drug cargos, during the preparation process of injection nanoformulations, was also reported [32,38,39]. The interactions between AGN Ex and RSV may play major roles in their loading into the NPs and their sustained release from NPs.

### 3.2. Particle Stability

**Figure 3:**
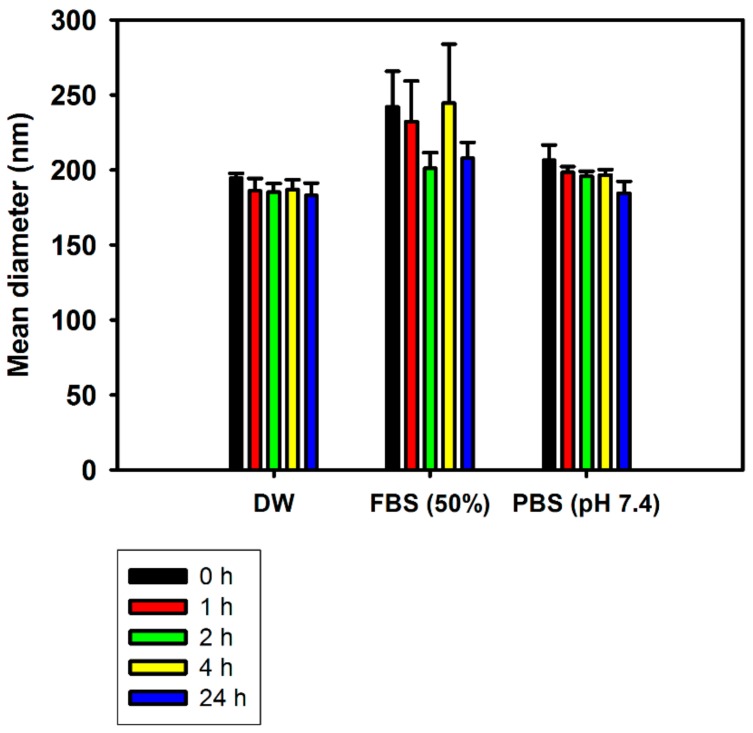
The particle stability of AGN/RSV NPs in DW, FBS (50%), and PBS (pH 7.4). Incubation time-dependent mean diameter values are plotted. Data are shown as the mean ± SD (*n* = 3).

The in vitro stability of AGN/RSV NPs was tested in different types of media (DW, FBS (50%), and PBS (pH 7.4)) simulating biological fluids (Figure 3). In DW, the AGN/RSV NPs exhibited constant mean diameters (183–195 nm) for 24 h. Although the hydrodynamic size of AGN/RSV NPs was slightly increased to 242 nm after mixing with FBS (50%) solution, it was reduced to 208 nm after a 24 h incubation. AGN/RSV NPs also exhibited the 184–206 nm mean diameters in PBS (pH 7.4) during a 24 h incubation. Generally, AGN/RSV NPs maintained their initial hydrodynamic size for 24 h in FBS (50%) solution as well as DW and PBS (pH 7.4). Those results imply that AGN/RSV NPs can maintain their nano-size even in the serum-included media as well as aqueous media (DW and PBS). It also suggests that AGN/RSV NPs may hardly form micro-size aggregates by interacting with blood components and prevent serious systemic toxicities (for example, embolization) after their intravenous administration [24]. In our previous report [24], AGN NPs also exhibited constant hydrodynamic sizes in DW, FBS (50%), and PBS (pH 7.4) for 24 h. The incorporation of RSV into the AGN NPs did not disturb the maintenance of initial particle size in the artificial biological media. The observed particle size range of AGN/RSV NPs in the aqueous media (DW and PBS) and serum-contained media may contribute to their efficient tumor targetability, which can be explained by the EPR effect.

### 3.3. Drug Release

**Figure 4:**
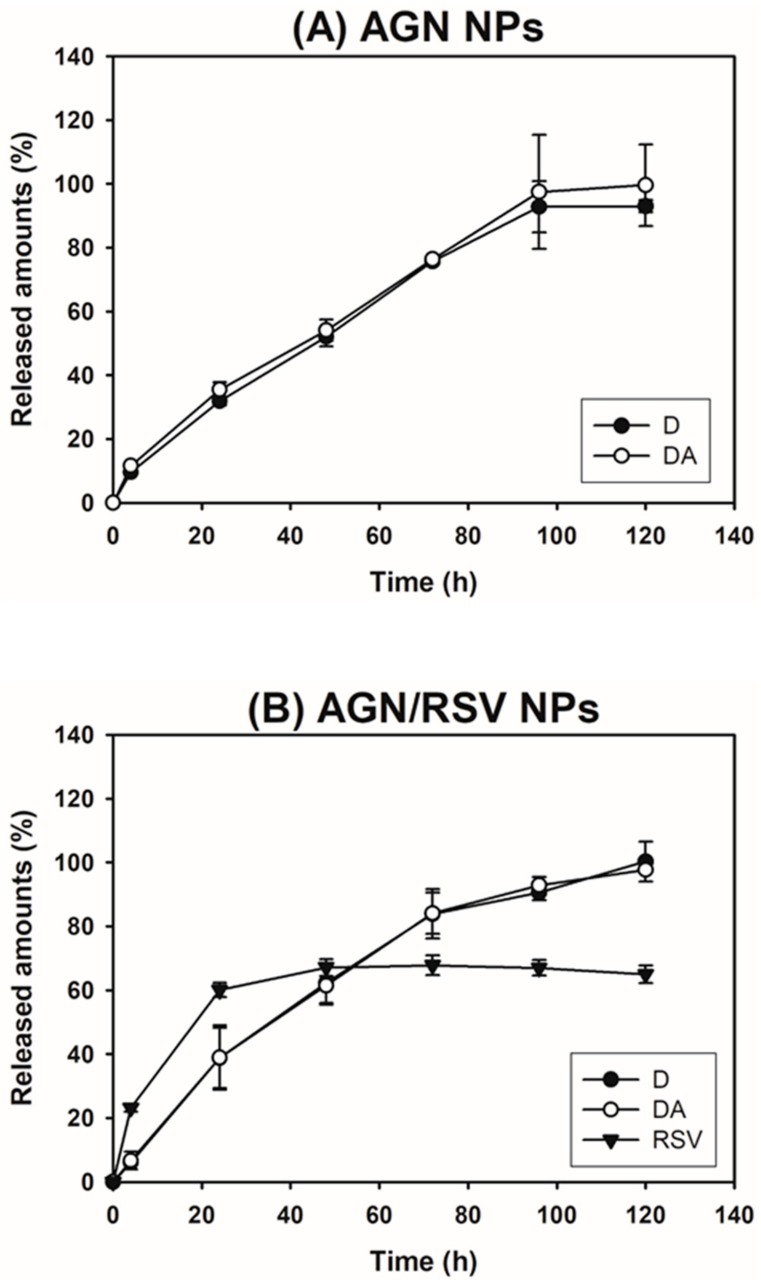
The release data of AGN NPs (D and DA) and AGN/RSV NPs (D, DA, and RSV). The cumulative released amounts of D, DA, and RSV from (**A**) AGN NPs and (**B**) AGN/RSV NPs are plotted. Data are shown as the mean ± SD (*n* = 3).

The release patterns of D, DA, and RSV from NPs in PBS were tested (Figure 4). D and DA were used as typical markers of AGN in the release test of our previous studies [24,26,27,28,29]. The cumulative percentage release of D and DA from AGN NPs at 24 h was 31.91 ± 1.34% and 35.44 ± 2.29%, respectively. They reached 92.95 ± 1.90% and 99.58 ± 12.77%, respectively, at 120 h. Those of D and DA from AGN NPs (prepared in this study) at 120 h were 2.8- and 3.4-fold higher than those reported data [24]. It might be due to the lower concentration of PVA (0.5%) used for the preparation of AGN NPs in this investigation compared to that (2%) in our previous report [24]. The cumulative percentage of released D and DA from AGN/RSV NPs at 24 h were 38.78 ± 9.38% and 38.90 ± 10.10%, respectively. Their values at 120 h were 100.22 ± 6.26% and 97.63 ± 0.47%, respectively. There was no significant difference between the released amounts of D and DA from AGN NPs and those from AGN/RSV NPs. The incorporation of RSV in NPs seems to hardly influence on the release profiles of D and DA from AGN/RSV NPs. The sustained release of RSV from AGN/RSV NPs was also observed in PBS (pH 7.4). The sustained drug release profiles can be considered as one of qualifications for the development of injection formulations. Sustained release of D, DA, and RSV from AGN/RSV NPs may reduce the dosing frequency and increase the patient compliance. The observed release patterns of active ingredients from AGN/RSV NPs may contribute to their efficient application to cancer therapy via intravenous route.

### 3.4. Cellular Uptake

**Figure 5:**
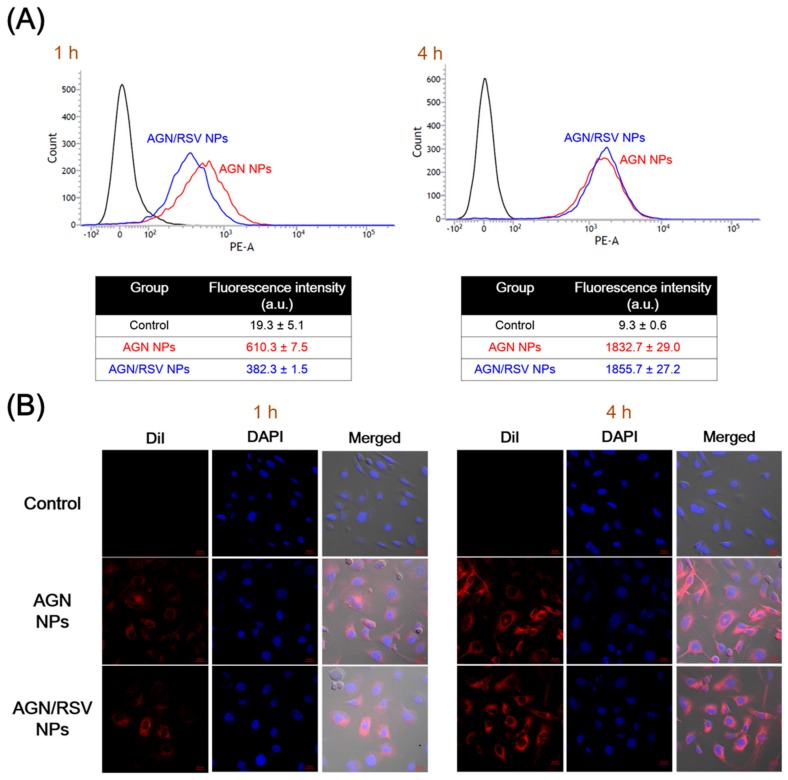
The cellular internalization and localization of DiI-loaded AGN NPs and AGN/RSV NPs in SKOV-3 cells. (**A**) The cellular internalization efficiencies of DiI-loaded NPs measured by flow cytometry. The mean fluorescence intensity values after 1 h and 4 h of incubation are presented. The data are shown as the mean ± SD (*n* = 3); (**B**) The intracellular localization of DiI-loaded NPs observed by CLSM imaging. DiI (red color), DAPI (blue color), and merged images of control, AGN NPs, and AGN/RSV NPs after 1 h and 4 h of incubation are shown. The length of scale bar (red color) is 20 μm.

The cellular internalization efficiency and intracellular location of AGN NPs and AGN/RSV NPs in SKOV-3 cells were studied by flow cytometry and CLSM imaging, respectively (Figure 5). DiI was encapsulated into the NPs as a fluorescent dye for measuring their cellular movement. Although the preparation method of AGN NPs was slightly different from that of our previous report [24], the released amount (6.4%) of DiI from AGN NPs after 24 h of incubation in DW implied that an amount of DiI was almost still entrapped in NPs during the incubation period for cellular uptake studies. Thus, it is expected that the fluorescence detection of DiI can estimate the cellular fate of DiI-loaded NPs. The SKOV-3 cell was used as a model cell line of ovarian cancers in this study.

The cellular internalization efficiency of AGN NPs and AGN/RSV NPs in SKOV-3 cells was assessed by flow cytometry analysis (Figure 5A). DiI-loaded AGN NPs and AGN/RSV NPs were incubated for 1 h and 4 h in SKOV-3 cells. After 1 h incubation, the average fluorescence intensity of AGN NPs-treated group was 1.60-fold higher than that of AGN/RSV NPs-treated group (*p* < 0.05). After 4 h of incubation, the average fluorescence intensity values of AGN NPs and AGN/RSV NPs were 3.00- and 4.85-fold higher than those values at 1 h, respectively (*p* < 0.05). There was no significant difference in the average fluorescence intensity values between AGN NPs and AGN/RSV NPs at 4 h. Although the cellular uptake rate of AGN/RSV NPs was lower than that of AGN NPs for 4 h, the final accumulated amounts of AGN NPs and AGN/RSV NPs at 4 h were similar. The intracellular distribution patterns of AGN NPs and AGN/RSV NPs also coincided with the data of flow cytometry analysis (Figure 5). Almost of NPs seemed to be located in the cytoplasm rather than the nucleus after their endocytosis (Figure 5B). All of these findings indicate that AGN/RSV NPs can show an equivalent cellular accumulation efficiency and similar intracellular localization compared to AGN NPs.

### 3.5. In Vitro Antiproliferation Efficiency

**Figure 6:**
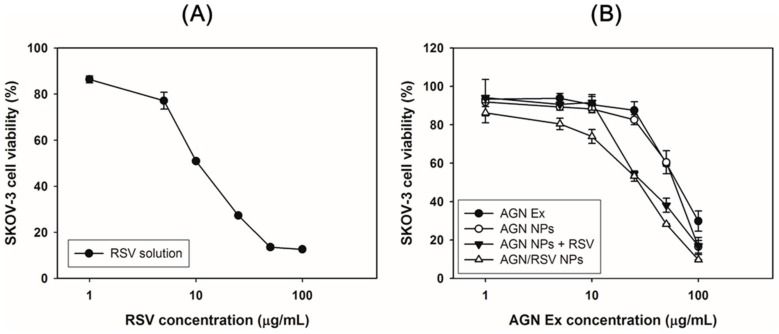
The antiproliferation data of RSV, AGN Ex, AGN NPs, AGN NPs + RSV, and AGN/RSV NPs in SKOV-3 cells. The cell viabilities according to RSV concentration (**A**) and AGN Ex concentration (**B**) are presented. The data are presented as the mean ± SD (*n* = 3).

The antiproliferation potentials of AGN/RSV NPs were evaluated in SKOV-3 cells by a colorimetric assay (Figure 6 and Table 2). The anticancer activities of ingredients and extracts of AGN against ovarian cancer cells were already reported somewhere [20]. In our previous report [24], the antiproliferation efficiency of AGN nanoformulation in breast cancer cells (that is, MCF-7 cells) was demonstrated. Notably, AGN Ex and AGN nanoformulation show negligible cytotoxicity below 50 μg/mL AGN Ex concentration in normal fibroblast (NIH3T3) cells [24]. It suggests that the AGN nanoformulation may have tumor-selective killing efficacies. RSV was incorporated into the AGN NPs to increase the therapeutic potentials for ovarian cancers. Its anticancer activities in ovarian cancer cells were already reported [40]. The antiproliferation efficiency of RSV in SKOV-3 cells was tested in this study (Figure 6A). The IC_50_ value of RSV in SKOV-3 cells after 72 h of incubation was 24.0 ± 1.5 μg/mL (Table 2). The antiproliferation potentials of AGN Ex (AGN Ex solution), AGN NPs, AGN NPs + RSV, and AGN/RSV NPs in SKOV-3 cells were evaluated by MTS-based assay (Figure 6B and Table 2). The IC_50_ values of AGN Ex, AGN NPs, AGN NPs + RSV, and AGN/RSV NPs were 70.1 ± 5.1 μg/mL, 60.0 ± 3.9 μg/mL, 53.4 ± 3.9 μg/mL, and 38.5 ± 2.1 μg/mL, respectively. The antiproliferation potentials of the experimental groups were ordered as follows: AGN Ex < AGN NPs AGN NPs + RSV < AGN/RSV NPs. The lower IC_50_ value of AGN/RSV NPs (*p* < 0.05), compared with that of AGN NPs, indicates that RSV can enhance the cytotoxicity in SKOV-3 cells in combination with AGN NPs. Notably, IC_50_ value of AGN/RSV NPs group was significantly lower than that of AGN NPs + RSV group (*p* < 0.05). It implies that dual loading strategy of AGN Ex and RSV in single nanocarrier can strengthen antiproliferation potentials via an efficient endocytosis. The observed antiproliferation results suggest that AGN/RSV NPs can provide improved anticancer activities in ovarian cancers after their arrival in tumor region via intravenous administration.

## 4. Conclusions

The RSV-incorporated AGN NPs were prepared by using emulsification and solvent evaporation methods. Dual anticancer agents (AGN Ex and RSV)-based NPs were fabricated by using the nanocrystal technology. AGN Ex acted as a pharmaceutical matrix of NPs as well as an anticancer agent. For improving anticancer potentials, RSV (as one of phytochemicals) was loaded to AGN NPs. The AGN/RSV NPs with 224 nm hydrodynamic size, <0.2 polydispersity index, and negative zeta potential values were developed. The spherical shape and narrow size distribution of AGN/RSV NPs were observed in the TEM image. Contrary to the crystalline properties of AGN Ex and RSV, both AGN NPs and AGN/RSV NPs exhibited amorphous states according to the results of XRD analysis. The initial hydrodynamic size of AGN/RSV NPs dispersion was maintained for 24 h in the serum-included media. The sustained release patterns of D, DA, and RSV from AGN/RSV NPs were observed at normal physiological pH (pH 7.4). In ovarian cancer (SKOV-3) cells, AGN/RSV NPs exhibited higher antiproliferation potentials rather than AGN Ex, AGN NPs, and AGN NPs + RSV. AGN/RSV NPs may be used as a prepotent nanosystem based on herbal extracts for the treatment of ovarian cancers.

## Figures and Tables

**Table 1:** The particle characterization of AGN NPs and AGN/RSV NPs (mean ± SD, *n* = 3).

Composition	Hydrodynamic Size (nm)	Polydispersity Index	Zeta Potential (mV)	Encapsulation Efficiency (%)
AGN NPs	221 ± 15	0.16 ± 0.02	−18.8 ± 0.4	47.95 ± 0.31 (AGN)
AGN/RSV NPs	224 ± 3	0.18 ± 0.01	−16.5 ± 1.8	46.97± 0.01 (AGN)
				49.22 ± 0.60 (RSV)

**Table 2:** IC_50_ values of RSV, AGN Ex, AGN NPs, AGN NPs + RSV, and AGN/RSV NPs in SKOV-3 cells.

Group	IC_50_ (µg/mL)
RSV	24.0 ± 1.5
AGN Ex	70.1 ± 5.1
AGN NPs	60.0 ± 3.9
AGN NPs + RSV	53.4 ± 3.9
AGN/RSV NPs	38.5 ± 2.1 *^,#,&^

IC_50_ values of AGN Ex, AGN NPs, AGN NPs + RSV, and AGN/RSV NPs groups are presented as AGN Ex concentration; Data are shown as the mean ± SD (*n* = 3); * *p* < 0.05, compared with AGN Ex group; ^#^ *p* < 0.05, compared with AGN NPs group; ^&^ *p* < 0.05, compared with AGN NPs + RSV group.
